# Draft Genome Sequences of Respiratory and Urinary Tract Isolates of *Acinetobacter baumannii* from the Same Patient

**DOI:** 10.1128/genomeA.00692-14

**Published:** 2014-07-17

**Authors:** Daniel Golemboski, Bertrand D. Eardly

**Affiliations:** a Department of Medical Laboratory Sciences, Bellarmine University, Louisville, Kentucky, USA; b Biology Department, Penn State Berks College, Reading, Pennsylvania, USA

## Abstract

*Acinetobacter baumannii* is a frequent hospital-acquired human pathogen. This report describes the draft genome sequences of two distinct *A. baumannii* clinical isolates from the same patient. A comparison of the genomes revealed differences in antibiotic resistance and will enable the determination of genomic differences responsible for virulence at each body site.

## GENOME ANNOUNCEMENT

The Gram-negative bacterium *Acinetobacter baumannii* has become increasingly relevant as both a community-acquired and a hospital-acquired pathogen (1, 2). The ability to survive under a variety of environmental conditions, including exposed dry surfaces, for extended periods of time make this typically low-level pathogen a ready candidate for widespread contamination. Outbreaks have been reported to result from *A. baumannii* contamination of medical equipment, in addition to most hospital surfaces (3–5). The respiratory and urinary tracts are among the most common sites of infection, which may be due in part to the hydrophobic nature of some strains conferring the ability to adhere to and colonize prosthetic devices, such as catheters and respiratory intubation tubing. Virulence mechanisms that have been attributed to *A. baumannii* include iron uptake (6, 7), adherence to epithelial cells (8), and biofilm formation (9).

Here, we present the draft genome sequences of two *A. baumannii* clinical isolates from the same patient but from different body sites. *A. baumannii* strain BU310 was initially isolated from a urinary catheter, followed by *A. baumannii* strain BR097 from a sputum specimen. Both strains demonstrated resistance to cefepime and tetracycline but differed in that BR097 is resistant to gentamycin while BU310 is susceptible. Similarly, BR097 was found to be resistant to ceftazidime, whereas BU310 demonstrated only intermediate resistance.

The genomes of both strains BR097 and BU310 were sequenced using the Illumina MiSeq platform with paired-end reads, resulting in mean genome coverages of 256-fold and 324-fold, respectively. The sequence reads were assembled using Velvet version 1.0.12 (10), resulting in 12 contigs for strain BR097 and 12 contigs for BU310. The genomes were annotated using the NCBI Prokaryotic Genomes Automatic Annotation Pipeline (PGAAP) (11). The mean G+C content of both strains is 38.93%. The annotation of strain BR097 predicted 3,629 protein-coding sequences (CDS), 18 copies of 5S, 16S, and 23S rRNA genes, and 63 tRNAs, whereas BU310 annotation predicted 3,601 CDS, 22 copies of rRNA genes, and 75 tRNAs.

An evaluation of these genomes using ResFinder (12) revealed that both strains possess resistance genes for aminoglycosides (*strA* and *strB*) and tetracycline [*tet*(B)]. The resistance genes *bla*_OXA-24_ and *bla*_OXA-95_, which confer β-lactam resistance, were detected in strain BR097; BU310 carries the *bla*_OXA-95_ gene, but it is missing *bla*_OXA-24_. The OXA-24 β-lactamase was shown to be involved in the carbapenem resistance of *A. baumannii* (13), which is consistent with these findings and with the antibiotic susceptibility testing that demonstrated resistance to imipenem by BR097 but not by BU310. Two prophages were found in each strain, using PHAST (14), which detected sequences identical to *Acinetobacter* phage Bphi-B1251 and *Psychrobacter* phage Psymv2. In addition, enterobacterial phage phiX174 was seen in strain BR097. In total, there are 134 phage genes in BU310 and 143 phage genes in BR097.

### Nucleotide sequence accession numbers.

This whole-genome shotgun project has been deposited at DDBJ/EMBL/GenBank under accession no. JNFY00000000 and JNFZ00000000. The versions described in this paper are versions JNFY01000000 and JNFZ01000000.
